# An exploratory survey on the state of training in adolescent medicine and health in 36 European countries

**DOI:** 10.1007/s00431-019-03445-1

**Published:** 2019-08-28

**Authors:** Pierre-André Michaud, Danielle Jansen, Lenneke Schrier, Johanna Vervoort, Annemieke Visser, Łukasz Dembiński

**Affiliations:** 1 University Hospital, Lausanne, Switzerland; 2 University Medical Centre Groningen, Department of Health Sciences, University of Groningen, Groningen, The Netherlands; 3 Willem-Alexander Children’s Hospital, Leiden University Medical Centre, Leiden, The Netherlands; 4 Department of Pediatric Gastroenterology and Nutrition, Medical University of Warsaw, Warsaw, Poland

**Keywords:** Adolescent, Adolescent medicine, Training, Medical education, Adolescent health, Europe, Survey

## Abstract

The development of adolescent health and medicine as a medical discipline lags behind in Europe compared with other regions of the world. This study aims to evaluate the structure and content of adolescent medicine and health training curricula for medical students, paediatricians, and other primary care physicians in the European region. A questionnaire survey was sent by e-mail to experts in the field from 36 European countries, addressing the content of adolescent health issues. Data was obtained from all 36 countries. At the undergraduate level, seven countries reported some mandatory stand-alone teaching (sessions dealing specifically with adolescents), while seven countries reported optional stand-alone teaching. In only 7 out of 36 countries were issues critical to adolescents covered as stand-alone sessions. At the postgraduate level, 15 countries delivered stand-alone mandatory training sessions to primary, secondary, or tertiary care paediatricians, covering most of the five critical areas listed in the questionnaire. In another 13 countries, such sessions were not mandatory and were inexistent in eight of them. The coverage among school physicians was similar but was much lower among general practitioners.

*Conclusion*: Paediatric associations and academic institutions should advocate for a better coverage of adolescent health and medicine in the training curricula of health care providers.

**Table:** 

**What is known:** • *In most European countries, adolescent medicine is still poorly represented as a discipline.* • *Experts have recently published recommendations regarding what form the structure and content of a training curriculum in this field should take.*	
**What is new:** • *This paper gives information on the extent and content of training in adolescent medicine and health as currently offered within under- and postgraduate European training curricula, in terms of stand-alone mandatory (*versus *optional) sessions.* • *In many European countries, both medical students and residents are poorly exposed to the basic knowledge and skills pertaining to adolescent health care.*

**What is known:**

• *In most European countries, adolescent medicine is still poorly represented as a discipline.*

• *Experts have recently published recommendations regarding what form the structure and content of a training curriculum in this field should take.*

**What is new:**

• *This paper gives information on the extent and content of training in adolescent medicine and health as currently offered within under- and postgraduate European training curricula, in terms of stand-alone mandatory (*versus *optional) sessions.*

• *In many European countries, both medical students and residents are poorly exposed to the basic knowledge and skills pertaining to adolescent health care.*

## Introduction

Worldwide, the specific health needs of adolescents are increasingly being addressed [13, 16, 18]. As mentioned in a recent publication of the World Health Organization (WHO) [23], “Investments in adolescent health bring a triple dividend of benefits for adolescents now, for their future adult lives, and for the next generation. Their health and well-being are engines of change in the drive to create healthier, more sustainable societies.” Policymakers and health professionals need to develop the technical capacity for policy, programming, research, and clinical care in all regions of the world. Several documents have recently outlined how high-quality health care can be achieved for adolescents [3, 15, 17, 22], in which the training and competencies of health care providers play a pivotal role [9, 12, 14]. When asked about their training needs in the area of adolescent medicine and health, paediatricians express a wish to acquire a variety of knowledge and skills. For instance, in a survey conducted among French paediatric residents, 81% considered that paediatricians should acquire skills in adolescent medicine and health; they reported major difficulties in providing care for teenagers reluctant to seek health care, or in managing suicidal adolescents [8]. In another survey carried out 15 years ago among Swiss primary care providers in private practice [10], two-thirds wanted to acquire more skills in managing functional disorders and half expressed a desire to receive training in areas such as communication skills, mental health (including eating disorders), substance use, or coping with dysfunctional families.

Training in the area of adolescent medicine and health needs encompasses more than just the delivery of knowledge; it also involves acquiring specific competencies and skills that allow the trainee to develop a mutually respectful relationship with an adolescent. Furthermore, it involves helping to develop appropriate screening and counselling approaches in reviewing the adolescent’s lifestyle, as well as learning how to deal with family conflicts and address situations posing ethical dilemmas. Finally, it means acquiring the capacity to deal with health issues such as exploratory and risk behaviours, mental health, and sexual and reproductive health as applied to adolescents10. This is why, in the opinion of the authors, it is insufficient to include some information on adolescent medicine within sessions dealing with broader areas; rather, *it is important to devote dedicated stand-alone* sessions to this field [12, 14]. Medical students and residents should be trained to deal concretely with clinical situations via interactive participative training sessions, bedside teaching and observation, and discussion of videos, and by testing out their skills with simulated patients [7, 12, 14]. In the USA, Canada, and Australia, and to a lesser extent in some South American nations, adolescent health and medicine is considered a medical discipline or sub-discipline, and most medical school and university hospitals provide both lectures and training sessions specifically addressing this population of patients [11, 16]. Compared with these countries, Europe is lagging behind [6, 11]. This study aims to evaluate the structure and content of adolescent training curricula in the European region for medical students, paediatricians, and other physicians who, regularly or occasionally, work as primary care providers, such as family physicians (GPs), gynaecologists, and psychiatrists. Specifically, it assesses the extent to which training in the field is provided as a stand-alone topic or embedded in the programmes of other disciplines, and whether it is mandatory or optional. A second objective is to explore the content of such training programmes in terms of issues that are most relevant to this period of life, such as communication skills, and issues pertaining to mental or sexual and reproductive health.

## Methods

This survey is part of the 4-year-long EU-funded MOCHA research programme (“Models of Child Health Appraised”), initiated by leading experts from Imperial College in London. This research programme aims to assess various aspects of primary care delivered to children and adolescents in EU countries, including topics such as quality assessment, economic factors, structure of health care delivery, training, and ethical aspects [4]. The overall project was undertaken by several specialist groups of researchers working in collaboration.

The responses to the questionnaire were provided by a MOCHA network of 30 experts, who, in each of their respective countries, were in charge of collecting data on various topics as a mandate of the MOCHA European project. To obtain as much information as possible, we used not only the MOCHA network but also other channels (Table 1). Thus, responses were gathered through two more sources: (1) paediatrician national delegates of the European Academy of Paediatrics (EAP) and (2) trainee representatives from “Young EAP” (e.g. paediatric residents and chief residents belonging to the Academy). The respondents from the MOCHA network were local experts in child health services, acting as informants for obtaining data requested by the scientists in charge of a given round; they used local indigenous sources to respond. These experts, one for each country, were selected for their expertise in child health services (paediatricians, psychologists, public health professionals) and were trained by the MOCHA project’s lead researchers. The respondents from the EAP were paediatricians, representatives of their country, while those answering from the Young EAP network were experienced paediatric residents or chief residents. All of these three groups’ respondents were instructed to do their best to gather valid data, e.g. asking colleagues and reviewing available documents. They all received the same questionnaire for collecting the data.

**Table 1:** List of the 36 countries involved, showing the sources of their answers

Armenia*	Germany*^#^	Norway*^#@^
Austria*^#@^	Greece*^#^	Poland*^#^
Belgium*^@^	Hungary*^#@^	Portugal*
Bosnia and Herzegovina*	Iceland*^#^	Romania^#^
Bulgaria*^#^	Ireland*^@^	Serbia*
Croatia*^#@^	Israel*	Slovenia*^#^
Cyprus*^#^	Italy*^#@^	Spain^#^
Czech Republic*^#^	Latvia^#@^	Sweden*^#@^
Denmark*	Lithuania*^#@^	Switzerland*^#^
Estonia*^#^	Malta^#@^	Turkey*
Finland*^#^	Moldova*	Ukraine
France*^#@^	Netherlands*^#@^	UK^#@^

*Information through the MOCHA network

^#^Information through the EPA network

^@^Information through the Young EPA network

The survey questionnaire was developed by the MOCHA group researching school and adolescent services, and was reviewed by colleagues from EAP and Young EAP. The content was based on a recent World Health Organization document: “Core competencies in adolescent health and development for primary care providers” [20]. Eleven questions concerned the structure and content of adolescent training curricula and pertained to different target learners, that is, students and residents of different disciplines. Given the fact that health care systems vary across countries (e.g. some systems train primary care paediatricians and others only secondary/tertiary care paediatricians), we decided to merge the information provided for these two groups. The questions evaluated whether the training sessions were *mandatory* or *optional*, and whether they were proposed as *stand-alone sessions* or were *embedded within the curriculum of various disciplines*. These questions did not address the existence of national curricula in adolescent medicine but pertained rather to whether training in this area did actually take place. A set of questions dealt with the content of these curricula or sessions, targeting five issues critical for adolescent health that were considered to be of particular importance by the working group [14, 19–21]: communication skills and counselling techniques as applied to adolescents, ethical and legal issues as applied to adolescents, screening of adolescent lifestyles and identification of risky behaviour, adolescent mental health, and adolescent sexual development and reproductive health. All the answers from the three groups were entered on a single comprehensive excel file and analysed manually. The annex provides the content of the questionnaire, as well as the instructions given to the experts. All questionnaires were administered in English, and the responses were provided in English as well. The survey took place during 2017 and 2018 and was sent out by email, and responses were collected via email too. One reminder was sent to those not responding to the first request. Most answers to the questions were proposed as yes/no or utilized a closed list of specific items (type of professional, location, etc.). Part of the answers are summarized in Tables 2 and 3. No statistical analysis was used. This survey was not submitted to an IRB, as it did not involve human subjects but dealt rather with procedures and policies.

**Table 2:** Number of countries that offer mandatory or optional training sessions within the postgraduate training of physicians in specific disciplines

Primary care paediatricians mandatory 11	
(Nonexistent in some countries) optional 3	
Not as a stand-alone 11	
Not applicable 11	
Primary care physicians (GPs) mandatory 3	
(Not applicable in some countries) optional 10	
Not as a stand-alone 20	
Not applicable 3	
Secondary care paediatricians mandatory 8	
(Not applicable in some countries) optional 12	
Not as a stand-alone 14	
Not applicable 2	
Gynaecologists mandatory 3	
Optional 18	
Not as a stand-alone 15	
Psychiatrists mandatory 9	
(Not applicable in some countries) optional 16	
Not as a stand-alone 11	
School physicians mandatory 10	
(Not applicable in some countries) optional 1	
Not as a stand-alone 5	
Not applicable 20

**Table 3:** Number of countries providing formal training in different areas within the postgraduate training curriculum of paediatricians and GPs (either as a stand-alone or embedded in other disciplines)

**Medical students:** • Communication skills: in 20 countries • Ethics in 22 countries • Screening of lifestyles in 21 countries • Sexual and reproductive health in 26 countries • Mental health in 25 countries	
**Paediatricians:** • Communication skills: in 21 countries • Ethics in 29 countries • Screening of lifestyles in 25 countries • Sexual and reproductive health in 22 countries • Mental health in 29 countries	
**General practitioners/family physicians:** • Communication skills: in 16 countries • Ethics in 18 countries • Screening of lifestyles in 16 countries • Sexual and reproductive health in 17 countries • Mental health in 23 countries	

For the majority of participating countries, data were received from more than one source (Table 1), i.e. for seven countries from three sources, for another 20 countries from two sources, and from nine countries from one source only. In some instances (not more than four to eight occasions, depending on the nature of the question), data available from different sources were only partially consistent. In cases where answers were inconsistent, the answer included in the final analysis was the one confirming that a training activity did indeed take place. This approach was based on the assumption that people who answered “yes” to the question knew of the existence/content of a training programme, while those who answered “no” may simply have not been aware of it. A complete set of answers (i.e. answers via either one or all of the three different sources) was received in 2017 or 2018 from all 36 European countries. Most questionnaires were adequately completed, with very few questions left unanswered (< 5%).

## Results

### Training in adolescent health and medicine for medical students

**Fig. 1:**
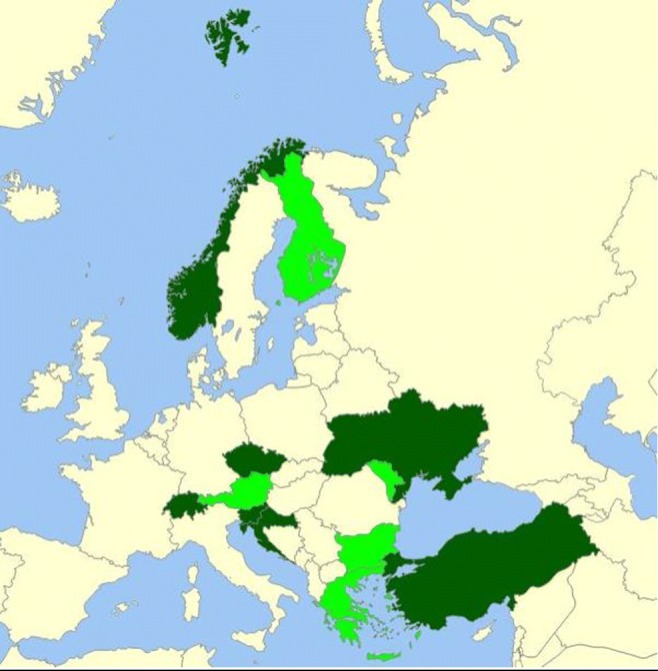
Countries providing stand-alone mandatory training sessions (dark green) or optional stand-alone sessions (light green) to medical students, irrespective of the content (*N* = 36 countries)

As shown in Fig. 1, representatives of seven countries reported that some *stand-alone* teaching was available and *mandatory* for medical students. These nations are Croatia, Czech Republic, Norway, Slovenia, Switzerland, Turkey, and Ukraine. In another set of seven countries, *optional stand-alone teaching* was provided: Austria, Bulgaria, Finland, Greece, Portugal, Moldova, and Spain. Twenty-two countries had no stand-alone programme for medical students. As shown in Table 3, a number of countries provided training (stand-alone/mandatory or not) tackling several specific adolescent health issues, but mostly as part of the programme of broader disciplines such as paediatrics, psychiatry, or gynaecology. Overall, in 19 countries, most of these issues were covered, and in another five, at least some were covered. In 12 countries, none of the subjects was covered as part of undergraduate studies: Austria, Bulgaria, Denmark, Greece, Hungary, Ireland, Israel, Italy, Latvia, Romania, Serbia, and Ukraine. Respondents were asked to gauge, among countries providing stand-alone adolescent medicine training sessions (*N* = 14), the percentage of medical schools that did so. In only three of them (Czech Republic, Norway, and Slovenia) was a figure of 75% or more reported; in the other countries, the figure was either lower than 30% or unknown.

### Training in adolescent health and medicine for residents

**Fig. 2:**
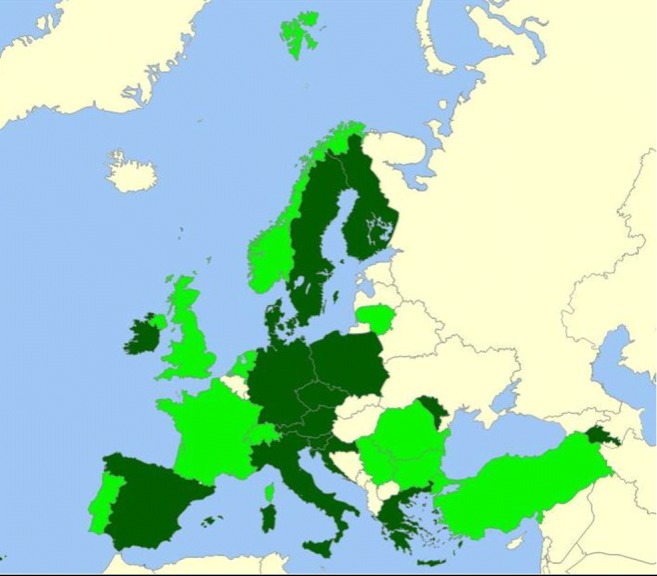
Countries providing stand-alone mandatory training sessions (dark green) or optional stand-alone sessions (light green) to residents in paediatrics, irrespective of the content (*N* = 36 countries)

Figure 2 shows that 15 countries provided mandatory stand-alone training sessions in the field of adolescent medicine and health to residents in paediatrics; 11 countries delivered stand-alone but optional courses, and ten countries did not offer any stand-alone education in this field. In other words (Table 2), a majority of the surveyed countries did not provide specific mandatory stand-alone sessions dealing with adolescent health to primary and secondary care paediatricians. In many instances, the sessions were optional and not necessarily stand-alone. This is even more apparent for family physicians: only three countries offered mandatory stand-alone courses to family physicians, and 14 included them as optional only. Comparable numbers were found for the training of gynaecologists and psychiatrists. Finally, ten out of 11 countries reported the existence of school health services involving physicians, thus including specific training to school doctors on a mandatory basis, but often not as stand-alone sessions.

Table 3 gives an overview of the adolescent health topics included within the postgraduate training of paediatricians and general practitioners (GPs). Mental health was the area covered most and in around three-fifths of the surveyed countries; the areas of communication skills, ethics, screening of lifestyles, and sexual and reproductive health were also included. The countries in which most of these topics were covered are Croatia, Finland, Iceland, Lithuania, Moldova, Norway, Slovenia, Sweden, Switzerland, Turkey, and the UK. In addition, several countries had established one or several specialised units where paediatric or internal medicine residents could acquire specific competencies from tutors trained in adolescent medicine. These were Croatia, Finland, France, Greece, Italy, Moldova, Portugal, Slovenia, Spain, Sweden, Switzerland, Turkey, and the UK.

In addition, in several countries there was some discrepancy between the format of training and its content. For instance, while Switzerland, Turkey, and Estonia offered a wide variety of topics to students, residents in paediatric and internal medicine (GPs), and to in-practice doctors, none of these was covered as stand-alone mandatory sessions. On the other hand, countries like Germany and Greece had implemented stand-alone training sessions in adolescent medicine and health but included only a limited number of topics in their curricula. Only a few countries such as Moldova or Finland provided training to paediatricians with mandatory stand-alone sessions covering all or most of the important topics.

### Training in adolescent health and medicine as part of continuing medical education

The percentage of countries organizing continuing medical education sessions in the field is fairly similar to that pertaining to postgraduate training. Fifteen countries offered such sessions in all or most of the topics considered important: Armenia, Austria, Bosnia and Herzegovina, Bulgaria, Croatia, Finland, France, Lithuania, Moldova, Romania, Slovenia, Spain, Sweden, Switzerland, and Turkey. The other countries (*N* = 11) did not offer a single session in the specific areas of adolescent health and medicine. Of interest is the fact that 17 countries (Austria, Croatia, Czech Republic, Denmark, Finland, France, Greece, Israel, Italy, Norway, Portugal, Slovenia, Spain, Sweden, Switzerland, Turkey, and the UK) had established an Association for Adolescent Medicine and Health, which may ultimately lead to a better range of offers at the CME level. The questionnaire did not allow assessment of the objectives and activities of these Associations.

## Discussion

Paediatricians and primary care providers (such as GPs) should acquire specific basic competencies in order to respond to the health care needs of adolescents [9, 12, 16]. In the USA, there are currently 26 accredited centres that offer CME programmes, residency to younger paediatricians or family doctors, as well as 2- or 3-year fellowships. Some of these centres have been in operation for more than 30 years. In addition, several academic institutions provide training programmes dealing with adolescent health for dieticians, psychologists, nursing students, or social workers. Australia also offers 4-year fellowship programmes in the field of “AYA” medicine (Adolescent and Young Adults). Both countries have created an official certified sub-specialisation in adolescent medicine. Some South America nations (Argentina, Chile, and Brazil) have their own academic centres. These academic centres have included areas in their educational programmes such as the acquisition of sound communication skills, expertise in dealing with sexual and reproductive health or substance misuse, and how to care for adolescents with chronic conditions; all these initiatives have an impact on the delivery of health care. While it is difficult to assess the impact of such training on the care of adolescents, a recent survey conducted in the USA shows that 43–81% of adolescents received provision of preventive services over the course of a year [1]. In contrast, such an achievement cannot be expected in the European region, given the lack of specific education in adolescent medicine and health within many training institutions [15, 21, 24]. Indeed, only seven out of the 36 surveyed European countries include mandatory stand-alone sessions in the undergraduate training of medical students, while in another seven countries, such sessions exist but are only optional. In several countries, residents in paediatrics receive training sessions covering important topics such as communication skills and ethics, screening of lifestyles, mental health, or sexual and reproductive health. However, in many instances, such training sessions are neither mandatory nor provided as stand-alone sessions. Moreover, it is likely that this type of education is not available in all regions of the countries in which they exist. The results regarding the training of general practitioners are of even greater concern: only three countries (< 10%) offer mandatory courses in the field.

A similar survey run on behalf of the EPA (European Paediatric Association—UNEPSA) [6] showed that in 2008, undergraduate education in adolescent medicine was offered in half of the countries surveyed (*N* = 14), and training in the field was provided in 18 out of 29 countries in the context of paediatric residency programmes. This earlier survey did not distinguish between mandatory and optional education and did not either investigate stand-alone teaching and learning. Given this situation, which seems not to have improved in the last 10 years, it comes as no surprise that, according to a recent UK survey, many adolescents feel dissatisfied with the provision of the health care they receive [25].

### Limitations

There are limitations to this survey. The most important one is that the respondents were usually not specialists in the field of adolescent health and medicine and came from different professional backgrounds. As a result, they may have not been able to obtain complete information about the state of training in this field, despite their efforts to gather as accurate information as possible. In addition, the respondents often mentioned that the distribution and organization of training curricula differed from one region or institution to another, but that it was not possible to measure to what extent this was the case. In some cases, the answers provided by respondents from different sources were not consistent. As we chose to keep the most positive answers, our findings may provide too optimistic a view of the situation within some countries. However, this problem does not jeopardize our conclusion as the situation may even be worse than the one we describe. Finally, the size of the questionnaires forced us to limit the number of topics included, as well as limit other details such as the number of hours delivered, or the types of teaching methods used. This survey must therefore be considered a first attempt to review the situation in Europe. In addition, for the same reason (questionnaire size), we were unable to include questions concerning the training of other professionals such as psychologists or social workers, despite the importance of an interprofessional approach in adolescent care, especially when dealing with complex situations such as long-term chronic conditions.

### Conclusion

What can be done to improve the situation? In our opinion, in the future, all European countries should endorse policies regarding adolescent-friendly primary care and the development of training sessions at under- and postgraduate level, as recommended recently by the Lancet commission, the World Health Organization, and various authors [2, 5, 9, 14, 16, 20, 21]. Apart from the development of policies, several bodies could contribute to the training of health professionals. A first step would be to introduce a few specific stand-alone mandatory training sessions pertaining to adolescent medicine and health in all medical faculties—and additionally in schools of nursing/midwifery [12]. The emphasis should be not only on specific knowledge regarding issues such as sexual and reproductive health, mental health, or substance use but also on attitudes to adopt when caring for adolescents, and skills such as communication skills. In this regard, an important step would be the systematic inclusion of topics related to adolescent medicine and health in the summative examinations using competency-based assessment approaches.

Most importantly, physicians planning to become paediatricians or GPs, as well as future gynaecologists and psychiatrists, should be the target of training initiatives at postgraduate level. In countries providing health care or preventive/screening activities within the school health setting, junior physicians could be exposed systematically, under supervision, to the specific needs of this young population. This also applies to outreach centres dealing with the vulnerable or dropout adolescent population. Ideally, the presence of a special ward or an adolescent outpatient clinic such as those created in London, Lisbon, or Lausanne allows for an effective sensitization of in-training residents. Academic institutions and university hospitals should ensure that one or several members of their paediatric staff develop an interest and specific competencies in the area of adolescent medicine and health, become credible mentors, and establish training programmes in the field. A typical content for such a curriculum—which should be created in collaboration with other disciplines—was recently proposed under the auspices of the European Academy of Paediatrics 11. Another useful tool is the EuTEACH programme (www.euteach.com), which provides a set of online training modules with slides and videos free of charge [9, 14]. In conclusion, a better coverage of adolescent health and medicine in the training curricula of medical students, residents in paediatrics, and other primary care disciplines is necessary. To achieve this, the voice and input of paediatric associations and academic institutions is needed.

## Abbreviations

EAP: European Academy of Paediatrics
EPA: European Paediatric Association
EU: European Union
EuTEACH: European training in effective adolescent care and health
GP: General practitioners
MOCHA: Models of Child Health Appraised
UK: United Kingdom
USA: United States of America
WHO: World Health Organization
